# Real‐World vs. Randomized Trial Evidence for Incretin‐Based Therapies: A Narrative Review

**DOI:** 10.1111/1753-0407.70248

**Published:** 2026-07-08

**Authors:** Zachary Bloomgarden

**Affiliations:** ^1^ Department of Medicine, Division of Endocrinology, Diabetes and Bone Disease Icahn School of Medicine at Mount Sinai New York New York USA

## Abstract

Incretin‐based therapies have transformed the management of obesity and type 2 diabetes (T2D). Pivotal randomized controlled trials (RCTs) report substantial weight loss and glycemic improvements; the extent to which these translate to clinical populations remains uncertain. Seven 2026‐published RCT meta‐analyses (comprising 127 RCTs and 58 976 participants) were compared with 26 real‐world studies identified through structured PubMed search. Primary outcomes were body weight loss and HbA1c reduction; secondary outcomes included major adverse cardiovascular events (MACE), neurological end‐points, and safety. The comparison is descriptive. For T2D, real‐world semaglutide achieved 4.7–10.5 kg weight loss (6–12 months) and 1.0–1.3 percentage‐point HbA1c reductions, lower than RCT meta‐analytic estimates (tirzepatide mean difference [MD] −9.55% vs. placebo). Without diabetes, real‐world persister analyses (SHAPE study: semaglutide −14.1%, tirzepatide −16.5%) approximated per‐protocol RCT estimates (semaglutide −14.9%, tirzepatide 15 mg −20.9%), whereas intention‐to‐treat real‐world estimates were substantially lower. Just 13% of semaglutide users reached 2.4 mg and 25.9% of tirzepatide users reached 15 mg. Real‐world cardiovascular data showed 20%–46% MACE reductions, broadly consistent with the RCT reduction. Differences between T2D and non‐T2D populations likely reflect channeling bias and differential follow‐up. Real‐world incretin‐based therapy effectiveness is broadly consistent with RCT efficacy when dose attainment is accounted for. High discontinuation rates (20%–50% within 1 year) and prior GLP‐1 RA exposure track with the observed efficacy gap. Clinicians can expect RCT‐level outcomes in patients who tolerate and persist with target doses. Head‐to‐head cardiovascular outcome trials comparing tirzepatide and semaglutide in non‐diabetic populations are needed.

## Highlights

- In randomized controlled trials, incretin‐based treatments have robust efficacy in reducing body weight and improving glycemic control, and have also been associated with improvement in cardiovascular and other clinically important outcomes.
- Clinicians, health systems, and policymakers remain uncertain about how well the strong results of carefully controlled RCTs translate to real‐world patient populations.
- The present narrative review compares the effects seen in meta‐analyses of RCTs to the results of real‐world effectiveness studies of incretin‐based therapies.

## Graphical abstract

- RCTs of incretin‐based treatment show effective weight loss and glycemic benefit, as well as improved CV outcomes.
- There is, however, uncertainty as to whether the RCTs translate to real‐world outcomes.
- This narrative review suggests real‐world outcomes are comparable to RCTs after accounting for submaximal dosing and high discontinuation rates.

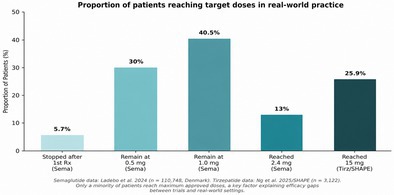

## 1 Introduction

The global prevalence of obesity exceeded 1 billion individuals in 2022 GBD 2021 Adult BMI Collaborators [1], while type 2 diabetes mellitus (T2D) affects approximately 537 million adults worldwide Genitsaridi [2], creating an unprecedented burden of cardiometabolic disease. In this context, incretin‐based therapies have emerged as a paradigm‐shifting class of medications. Glucagon‐like peptide‐1 receptor agonists (GLP‐1 RAs) such as semaglutide and liraglutide, along with the dual GLP‐1/glucose‐dependent insulinotropic polypeptide (GIP) receptor agonist tirzepatide, have demonstrated robust efficacy in reducing body weight and improving glycemic control in pivotal randomized controlled trials (RCTs) [3, 4, 5].

The STEP program established subcutaneous semaglutide 2.4 mg as a potent anti‐obesity agent, with the STEP 1 trial reporting a mean placebo‐subtracted weight reduction of approximately 12.4% over 68 weeks in adults without diabetes [3]. The SURMOUNT program subsequently demonstrated even greater efficacy for tirzepatide, with the highest dose (15 mg) producing approximately 20.9% weight loss versus placebo in people without T2D [4]. In people with T2D, the SURPASS trials showed that tirzepatide achieved superior HbA1c reduction (up to 2.3 percentage points) and weight loss compared with semaglutide 1.0 mg [5]. Beyond metabolic end‐points, the SELECT trial (semaglutide 2.4 mg, *n* = 17 604) demonstrated a 20% reduction in major adverse cardiovascular events (MACE) in people with obesity but without diabetes, establishing cardiovascular benefit independent of glycemic control [6].

These findings have catalyzed rapid adoption of incretin‐based therapies globally. However, a fundamental question confronts clinicians, health systems, and policymakers: to what extent do the impressive effect sizes observed in carefully controlled RCTs translate to heterogeneous real‐world patient populations? This question is not merely academic. RCTs enroll highly selected participants who undergo intensive dose titration with close monitoring, whereas real‐world patients often have multiple comorbidities, variable adherence, suboptimal dose escalation, and limited specialist oversight. Understanding the magnitude and drivers of any efficacy–effectiveness gap is essential for evidence‐based prescribing, resource allocation, and patient counseling.

Several factors may contribute to discordance between RCT and real‐world outcomes. First, dose titration behavior differs markedly: a Danish nationwide registry study of 110 748 semaglutide users found that only 13% reached the maximum approved dose of 2.4 mg, with the largest proportion (33%–48%) remaining at 1.0 mg from the fourth prescription onward [7]. Second, real‐world populations include patients with prior GLP‐1 RA exposure, who may exhibit attenuated responses compared with treatment‐naïve individuals. Third, high discontinuation rates—estimated at 20%–50% within the first year—reduce the effective treatment duration relative to RCTs [8]. Fourth, industry sponsorship of clinical trials (96.2% of GLP‐1 RA weight loss RCTs according to a 2026 meta‐analysis) raises questions about the generalizability of trial populations and the potential for publication bias [9].

In 2026, a wave of large‐scale meta‐analyses synthesized the RCT evidence base for incretin‐based therapies, providing robust estimates of expected efficacy under controlled conditions. Simultaneously, a growing body of real‐world evidence—from electronic health records, registries, and target trial emulations—has accumulated, for the first time enabling systematic comparison of trial and practice‐based outcomes.

This narrative review addresses the following research question: Do the results of real‐world effectiveness studies of incretin‐based therapies for weight loss and HbA1c reduction match the effect sizes seen in RCT meta‐analyses, and where do meaningful differences emerge, separately in people having and not having type 2 diabetes? The RCT meta‐analyses are compared with 26 real‐world studies to quantify the efficacy–effectiveness gap, identify its drivers, and examine secondary outcomes including cardiovascular events, neurological end‐points, and safety.

## 2 Methods

### 2.1 Study Design and Data Sources

This is a systematic descriptive comparison of RCT meta‐analytic evidence with real‐world effectiveness studies. No new meta‐analysis was performed. Two complementary search strategies were employed.

For real‐world evidence, a structured PubMed search was conducted in April 2026 using the following strategy: “real world” [ti] combined with drug‐specific terms (semaglutide, tirzepatide, liraglutide, dulaglutide, exenatide, lixisenatide, GLP‐1, incretin). This search identified 26 studies meeting inclusion criteria, including observational cohort studies, target trial emulations, propensity‐score matched analyses, and registry‐based studies. For RCT evidence, seven meta‐analyses published in 2026 were identified through targeted searching of JAMA Internal Medicine, Obesity, Advances in Therapy, Diabetes Obesity and Metabolism, Cureus, and Frontiers in Endocrinology. These meta‐analyses collectively synthesized data from up to 127 RCTs involving 58 976 participants (Table 1) [9, 10, 11, 12, 13, 14, 15].

**TABLE 1:** Summary of meta‐analyses of RCTs included in this review.

References	Year	Journal	Scope	*N* (RCTs/Pts)	Population	Primary outcome	Key finding
Lim et al.	2026	Obesity	NMA, weight	15/14 059	Non‐T2D	% weight loss	Tirz 15 mg −20.9%; Sema 2.4 mg −14.9% vs. placebo
Ciudin et al.^a^	2026	Adv Ther	Bayesian NMA	6/NR	Non‐T2D	% weight loss	Tirz 15 mg −6.26% more than sema 2.4 mg
Vienghirun et al.	2026	Diab Obes Metab	NMA	127/58 976	T2D + non‐T2D	Weight loss, ≥ 5%/≥ 10%	Tirz MD −9.55% (T2D), −16.48% (non‐T2D) vs. placebo
Tom‐Ayegunle et al.	2026	Cureus	MA, CV outcomes	16/23 467	Non‐T2D obesity	MACE	GLP‐1 RA: RR 0.80 (0.72–0.89) for MACE
Alexander et al.	2026	JAMA Intern Med	HTE MA	64/NR	Mixed	Weight loss HTE	Women −10.9% vs. men −6.8%; no HTE by age, race, BMI
Mirghani et al.	2026	Front Endocrinol	MA, T1D	18/3157	T1D	HbA1c, weight	HbA1c −0.4%; weight −4.28 kg; AEs higher
Tahir et al.	2026	Diab Obes Metab	MA, GLP‐1 vs. BS	15/20 594	Obesity	Weight loss	BS superior > 1 year (MD −19.78 kg); comparable at 6 months

Abbreviations: BS, bariatric surgery; HTE, heterogeneity of treatment effects; MA, meta‐analysis; NMA, network meta‐analysis; NR, not reported; Pts, participants; Sema, semaglutide; Tirz, tirzepatide.

^a^ Industry‐sponsored: funded by Eli Lilly and Company, manufacturer of tirzepatide.

### 2.2 Population Stratification

Studies were stratified by diabetes status: (1) people with T2D, (2) people without T2D (overweight/obesity), and (3) people with type 1 diabetes (T1D). This stratification was applied because both the magnitude of weight loss and the mechanisms of cardiovascular benefit differ substantially between these populations, with RCT meta‐analyses consistently reporting greater percentage weight loss in non‐diabetic populations [11].

### 2.3 Outcome Measures

Primary outcomes were body weight loss (reported as both kilograms and percentage of baseline body weight where available) and HbA1c reduction (percentage points). Secondary outcomes included MACE (composite of cardiovascular death, non‐fatal myocardial infarction, and non‐fatal stroke), individual cardiovascular end‐points, neurological outcomes, pulmonary outcomes, psychiatric outcomes, and safety (adverse events, discontinuation rates).

### 2.4 Evidence Quality Assessment

Real‐world studies were categorized by evidence quality adapting the approaches of the GRADE guidelines (Balshem et al. [16]) and of the Agency for Healthcare Research and Quality recommendations for assessing Comparative Effectiveness Reviews (Owens et al. [17]). High‐quality evidence included large, peer‐reviewed studies employing robust causal‐inference methods such as target trial emulation, propensity‐score matching, or comparable approaches to confounder control. Moderate‐quality evidence included adequately powered observational studies with appropriate statistical adjustment for key confounders. Evidence was ranked of low‐quality if the sample size was small, representing a single center, or with limited adjustment for confounders. All meta‐analyses were assessed for potential bias, including industry sponsorship. Notably, 96.2% of RCTs included in the Alexander et al. heterogeneity of treatment effects (HTE) meta‐analysis were industry‐funded [9]. The Ciudin et al. Bayesian NMA was funded by Eli Lilly and Company, the manufacturer of tirzepatide, and excluded the SURMOUNT‐5 head‐to‐head trial on methodological grounds that merit scrutiny [13].

### 2.5 Statistical Approach

This study employs descriptive comparison rather than quantitative synthesis. Effect sizes from RCT meta‐analyses (reported as mean differences, risk ratios, or hazard ratios with 95% confidence intervals) were compared qualitatively and quantitatively with corresponding real‐world estimates. Where multiple real‐world studies reported the same outcome, the range of estimates is presented. No formal statistical test of heterogeneity between RCT and real‐world estimates was performed; instead, the magnitude and direction of discordance are described and potential explanations discussed.

## 3 Results

### 3.1 Weight Loss in People With Type 2 Diabetes

Real‐world studies of semaglutide in people with T2D consistently reported weight reductions of 4.7–10.5 kg over 6–12 months of treatment (Table 2). In the SURE Netherlands observational study (*n* = 186, 30 weeks), once‐weekly semaglutide produced a mean weight loss of 7.8 kg (7.5% of baseline body weight), accompanied by a 1.2 percentage‐point reduction in HbA1c [18]. Thewjitcharoen et al. reported a more modest 4.7 kg reduction in a Thai cohort (*n* = 58, 6 months) [19], while Alkhalifah et al. observed 10.5 kg at 12 months in a US cohort (*n* = 106) using mixed semaglutide formulations [20]. In Italy, Marzullo et al. found that only 21%–25% of patients achieved a clinically meaningful threshold of ≥ 5% weight loss, and 7%–18% achieved ≥ 10% [21].

**TABLE 2:** Weight loss: RCT meta‐analyses vs. real‐world studies—people with type 2 diabetes.

Study	Type	Drug	*N*	Follow‐up	Wt loss (kg)	Wt loss (%)	Evidence
RCT meta‐analyses							
Vienghirun 2026	NMA	Tirzepatide	58 976^a^	24–52 weeks	NR	−9.55% vs. PBO	HIGH (127 RCTs)
Vienghirun 2026	NMA	Sema SC	58 976^a^	24–52 weeks	NR	~5%–6% vs. PBO	HIGH
Real‐world studies							
Wolffenbuttel 2023	Obs	Sema SC	186	30 weeks	−7.8	−7.5%	MODERATE
Thewjitcharoen 2023	Obs	Sema SC	58	6 months	−4.7	NR	LOW
Alkhalifah 2024	Obs	Sema mixed	106	12 months	−10.5	~10%	MODERATE
Buckley 2024	Retro	Tirz overall	3686	40 weeks	−4.5	−4.8%	HIGH
Buckley 2024	Retro	Tirz (naïve)	—	40 weeks	−7.2	NR	HIGH

Abbreviations: NR, not reported; Obs, observational; PBO, placebo; Retro, retrospective; SC, subcutaneous; Sema, semaglutide; Tirz, tirzepatide.

^a^ Total participants across all arms and populations in the Vienghirun NMA.

For tirzepatide in T2D, Buckley et al. reported a mean weight loss of 4.5 kg (4.8%) at 40 weeks in a large UAE healthcare system cohort (*n* = 3686) [22]. Importantly, GLP‐1 RA‐naïve patients achieved substantially greater weight loss (7.2 kg) than those with prior GLP‐1 RA exposure, suggesting that treatment history is a key modifier of real‐world effectiveness. A large‐scale comparison by Chaiyakunapruk et al. (*n* = 31 111) found that the combination of GLP‐1 RA plus SGLT2 inhibitor produced greater weight reduction than SGLT2 inhibitor alone, with once‐weekly semaglutide showing the highest weight loss in the GLP‐1 RA class [23].

These real‐world estimates fall below RCT meta‐analytic benchmarks. The Vienghirun NMA (127 RCTs, *n* = 58 976) reported tirzepatide MD −9.55% (95% CI −10.94 to −8.15) versus placebo in people with T2D, with a SUCRA ranking of 100 (highest efficacy) [11]. The relative likelihood for achieving ≥ 5% weight loss was 7.17 (95% CI 4.38–11.73) for tirzepatide and 4.74 (95% CI 3.17–7.08) for subcutaneous semaglutide. For the more stringent threshold of ≥ 10% weight loss, tirzepatide achieved likelihood 14.34 (95% CI 5.98–34.35) and semaglutide likelihood 6.12 (95% CI 2.97–12.59).

The comparison with bariatric surgery provides additional context. Tahir et al. (15 studies, *n* = 20 594) found that GLP‐1 RA and bariatric surgery did not differ significantly in weight loss at 6 months (MD −12.19 kg; *p* = 0.13), although bariatric surgery was superior at ≤ 1 year (MD −16.97 kg; *p* = 0.02) and beyond 1 year (MD −19.78 kg; *p* < 0.001), albeit with high heterogeneity (*I* ^2^ = 74%–99%) [14].

Three factors principally account for the RCT–real‐world gap in T2D. First, sub‐maximal dosing: Ladebo et al. found that in 110 748 Danish semaglutide users, only 13% ever reached the maximum dose of 2.4 mg, with 5.7% discontinuing after the first prescription and 33%–48% remaining at 1.0 mg from the fourth prescription onward [7]. Second, prior GLP‐1 RA exposure attenuates responses, as demonstrated by the 2.7 kg difference between GLP‐1 RA‐naïve and experienced patients in the Buckley tirzepatide cohort [22]. Third, real‐world populations are less selected than RCT participants, with higher burdens of comorbidity, polypharmacy, and variable monitoring intensity.

### 3.2 HbA1c Reduction in People With Type 2 Diabetes and Type 1 Diabetes

Real‐world HbA1c reductions in T2D were remarkably consistent across studies, ranging from 0.6 to 1.3 percentage points (Table 3). Thewjitcharoen et al. reported a 1.3 percentage‐point reduction with once‐weekly semaglutide [19], Wolffenbuttel et al. observed 1.2 percentage points in the SURE study [18], and Alkhalifah et al. found 1.4 percentage points at 12 months [20]. The lowest estimate came from Buckley et al. (tirzepatide, −0.6 percentage points overall; −1.0 in GLP‐1 RA‐naïve patients), again demonstrating the attenuation associated with prior incretin exposure [22].

**TABLE 3:** Real world HbA1c reduction: T2D and T1D.

Study	Type	Drug	Pop.	*N*	Follow‐up	HbA1c Δ (%‐pts)	Notes
Real‐world—T2D							
Wolffenbuttel 2023	Obs	Sema OW	T2D	186	30 weeks	−1.2	SURE Netherlands
Marzullo 2022	Obs	Sema OW	T2D	154	Variable	−1.02	Italy; ≥ 5% wt loss 21%–25%
Thewjitcharoen 2023	Obs	Sema OW	T2D	58	6 months	−1.3	Thailand
Alkhalifah 2024	Obs	Sema mixed	T2D	106	12 months	−1.4	US
Buckley 2024	Retro	Tirzepatide	T2D	3686	40 weeks	−0.6 (−1.0 naïve)	UAE; prior GLP‐1RA attenuates
Real‐world—T1D							
Mirghani 2026	MA	GLP‐1 RA (liraglutide)	T1D	3157	Variable	−0.4%	*I* ^2^ = 100%; *p* = 0.03; AEs higher

*Note:* See Tables 2 and 4 for other abbreviations.

Abbreviations: %‐pts, percentage points; AEs, adverse events; Pop., population; Δ, change.

These real‐world HbA1c reductions are generally consistent with, though at the lower end of, RCT expectations. The SURPASS trials reported tirzepatide‐associated HbA1c reductions of approximately 1.5–2.3 percentage points depending on dose and comparator [5], and semaglutide trials in T2D typically report 1.0–1.8 percentage‐point reductions. The narrower gap for HbA1c compared with weight loss may imply that glycemic control is less dose dependent above a threshold dose.

In T1D, the Mirghani et al. meta‐analysis (18 studies, *n* = 3157) found a modest HbA1c reduction with GLP‐1 RAs (MD −0.4 percentage points; 95% CI −0.77 to −0.03; *p* = 0.03), although heterogeneity was extreme (*I* ^2^ = 100%) [15]. Weight reduction was more robust (MD −4.28 kg; 95% CI −5.06 to −3.49). Critically, GLP‐1 RAs did not increase hypoglycemia (MD 0.08; 95% CI −0.88 to 1.04) or alter C‐peptide levels, but total adverse events were significantly higher. The most commonly studied agent was liraglutide; data for semaglutide and tirzepatide in T1D are extremely limited.

### 3.3 Weight Loss in People Without Diabetes

Real‐world weight loss data in people without T2D showed a striking pattern dependent on the analytic approach (Table 4). The SHAPE study (Ng et al. 2025) reported that among persistent users (*n* = 9916), semaglutide 2.4 mg produced 14.6 kg (14.1%) weight loss and tirzepatide produced 17.2 kg (16.5%) at 12 months [24]. In contrast, an intention‐to‐treat analysis by Anson et al. (*n* = 13 846, including patients who discontinued treatment) found substantially lower estimates: tirzepatide −7.7 kg versus semaglutide −4.8 kg (*p* < 0.001), assessed at 12 months after treatment cessation [25]. A small single‐center UAE study (Bhatti et al., *n* = 269) reported even more divergent results: semaglutide −11.6% versus tirzepatide −22.0% at 12 months, though these findings are limited by small sample size and possible selection bias [26].

**TABLE 4:** Weight loss: RCT meta‐analyses vs. real‐world studies—people without type 2 diabetes.

Study	Type	Drug	*N*	Follow‐up	Wt loss (kg)	Wt loss (%)	Evidence
RCT meta‐analyses							
Lim 2026	NMA	Tirz 15 mg	14 059^b^	Variable	NR	−20.9% vs. PBO	HIGH (15 RCTs)
Lim 2026	NMA	Tirz 10 mg	14 059^b^	Variable	NR	−17.8% vs. PBO	HIGH
Lim 2026	NMA	Sema 2.4 mg	14 059^b^	Variable	NR	−14.9% vs. PBO	HIGH
Vienghirun 2026	NMA	Tirz (all)	58 976^b^	24–52 weeks	NR	−16.48% vs. PBO	HIGH
Ciudin 2026^a^	NMA	Tirz 15 mg vs. Sema	NR	Variable	NR	−6.26% addtl	HIGH (industry)
Real‐world studies							
SHAPE/Ng 2025	Persisters	Sema 2.4 mg	9916	12 months	−14.6	−14.1%	HIGH
SHAPE/Ng 2025	Persisters	Tirzepatide	9916	12 months	−17.2	−16.5%	HIGH
Anson 2024	Off‐drug ITT	Tirzepatide	13 846	12 months	−7.7	~7%	HIGH
Anson 2024	Off‐drug ITT	Semaglutide	13 846	12 months	−4.8	~4.5%	HIGH
Bhatti 2025	Obs	Tirz	269	12 months	NR	−22.0%	LOW
Bhatti 2025	Obs	Sema	269	12 months	NR	−11.6%	LOW

*Note:* See Table 2 for other abbreviations.

Abbreviations: addtl, additional; ITT, intention‐to‐treat.

^a^ Industry‐sponsored: funded by Eli Lilly and Company.

^b^ Total participants across NMA.

The Lim NMA (15 RCTs, *n* = 14 059, non‐diabetic adults) provides the key RCT benchmark. Placebo‐adjusted weight reductions were: tirzepatide 15 mg −20.9%, tirzepatide 10 mg −17.8%, semaglutide 2.4 mg −14.9%, and liraglutide 3 mg −8.0% [12]. The Vienghirun NMA reported tirzepatide MD −16.48% (95% CI −21.70 to −11.27) versus placebo in non‐T2D populations [11]. The Ciudin/Eli Lilly Bayesian NMA estimated tirzepatide 15 mg to produce 6.26 additional percentage points of weight loss beyond semaglutide 2.4 mg, and 13.95 additional points beyond liraglutide [13].

The real‐world persister analysis from SHAPE closely approximates per‐protocol RCT estimates: semaglutide −14.1% (SHAPE) versus −14.9% (Lim NMA), and tirzepatide −16.5% (SHAPE) versus −20.9% at 15 mg and −17.8% at 10 mg (Lim NMA). Since only 25.9% of SHAPE tirzepatide users reached 15 mg, the −16.5% figure aligns well with the expected dose‐weighted average. In contrast, intention‐to‐treat real‐world estimates (Anson 2024: approximately 7% for tirzepatide, 4.5% for semaglutide) are roughly half the RCT estimates, underscoring the impact of discontinuation and dose non‐attainment [7, 24].

The Alexander et al. HTE meta‐analysis (41 articles, 64 RCTs) identified a significant sex difference in GLP‐1 RA efficacy: women achieved 10.9% (95% CI 7.0%–14.8%) weight loss versus 6.8% (95% CI 4.6%–9.0%) for men [9]. This sex disparity has not yet been systematically assessed in real‐world studies and represents an important gap. No significant heterogeneity of treatment effects was detected by age, race, ethnicity, baseline BMI, or baseline HbA1c. Of note, tirzepatide was excluded from this meta‐analysis because it is a dual agonist rather than a pure GLP‐1 RA, limiting the applicability of these HTE findings to the dual agonist class.

### 3.4 Cardiovascular Outcomes

Cardiovascular outcome data reveal a complex picture with an intriguing paradox between T2D and non‐T2D populations (Table 5).

**TABLE 5:** Cardiovascular outcomes: RCT vs. real‐world.

Study	Type	Drug/Comp.	Pop.	*N*	Follow‐up	CV outcome	Effect (95% CI)	Strength
RCT meta‐analyses								
Tom‐Ayegunle 2026	MA	GLP‐1RA vs. PBO	Non‐T2D	23 467	68 weeks med	MACE‐3	RR 0.80 (0.72–0.89)	HIGH
Tom‐Ayegunle 2026	MA	GLP‐1RA vs. PBO	Non‐T2D	23 467	68 weeks med	Stroke	RR 0.72	HIGH
Tom‐Ayegunle 2026	MA	GLP‐1RA vs. PBO	Non‐T2D	23 467	68 weeks med	MI	RR 0.84	HIGH
Tom‐Ayegunle 2026	MA	GLP‐1RA vs. PBO	Non‐T2D	23 467	68 weeks med	CV Death	RR 0.85 (0.73–0.99)	HIGH
Tom‐Ayegunle 2026	MA	GLP‐1RA vs. PBO	Non‐T2D	23 467	68 weeks med	Total Mortality	RR 0.88 (0.78–0.99)	HIGH
Real‐world studies								
Chaiyakunapruk 2025	RW	GLP‐1RA + SGLT2i vs. SGLT2i	T2D	31 111	NR	MACE‐3	HR 0.54	HIGH
Anson 2024	RW	Tirz vs. Sema	T2D	8446	NR	Composite CV	HR 0.54 (0.38–0.76)	HIGH
Henney 2025	RW	Tirz vs. Sema	T2D + OSA	NR	NR	MACE	HR 0.86 (0.74–0.99)	HIGH
Wilson/STEER 2026	RW	Sema vs. Tirz	Non‐T2D ASCVD	21 250	NR	rMACE‐3	HR 0.71 (*p* = 0.046)	HIGH
Augusto 2025	RW	Tirz vs. no Tx	HF no T2D	1794	NR	MACE	HR 3.57 fav. tirz	MODERATE

*Note:* See Tables 2 and 4 for other abbreviations.

Abbreviations: ASCVD, atherosclerotic cardiovascular disease; Comp., comparison; CV, cardiovascular; fav., favoring; HF, heart failure; med, median; Tx, treatment.

In people with T2D, real‐world studies consistently showed cardiovascular benefit favoring incretin‐based therapies over active comparators. Chaiyakunapruk et al. (*n* = 31 111) reported that the combination of GLP‐1 RA plus SGLT2 inhibitor versus SGLT2 inhibitor alone was associated with HR 0.54 for 3‐point MACE, HR 0.58 for stroke, and HR 0.63 for myocardial infarction [23]. Anson et al. compared tirzepatide with semaglutide in T2D and found HR 0.54 (95% CI 0.38–0.76) for composite cardiovascular events and HR 0.33 (95% CI 0.15–0.73) for all‐cause mortality, favoring tirzepatide [25]. Henney et al., in a T2D cohort with obstructive sleep apnea, reported that tirzepatide was associated with lower MACE risk than both liraglutide (HR 0.58; 95% CI 0.51–0.66) and semaglutide (HR 0.86; 95% CI 0.74–0.99) [27]. RCT data from SURPASS‐CVOT confirm tirzepatide's cardiovascular benefit versus dulaglutide in T2D with established cardiovascular disease (HR 0.85 for 3‐point MACE), with post hoc analyses showing additional cardiorenal benefits [28, 29]. An imputed placebo analysis of SURPASS‐CVOT yielded an estimated MACE risk ratio of 0.80 versus placebo, aligning with semaglutide's SELECT trial estimate [30]. Network meta‐analyses confirm tirzepatide's cardiovascular superiority versus GLP‐1 RAs as a class in T2D [31]. In T2D, semaglutide's cardiovascular benefit is further supported by SUSTAIN 6 and PIONEER 6 trial data showing MACE reductions of approximately 26% and 21% respectively [32, 33]. A large active‐comparator real‐world study (Kruger et al., *n* = 113 209 T2D patients) found no significant difference in 12‐month MACE rates between semaglutide and tirzepatide (HR 0.97; 95% CI 0.88–1.07), supporting class‐level rather than drug‐specific cardiovascular benefit in T2D clinical practice [34].

In people without T2D, the pattern reversed. The STEER study by Wilson et al. (*n* = 21 250, non‐T2D with established atherosclerotic cardiovascular disease) found that semaglutide was associated with lower recurrent MACE‐3 compared with tirzepatide (HR 0.71; *p* = 0.046), a finding that strengthened in the per‐protocol analysis (HR 0.43; *p* = 0.005) [35]. This aligns directionally with the Tom‐Ayegunle RCT meta‐analysis (16 RCTs, *n* = 23 467, non‐T2D obesity), which reported a 20% MACE reduction (RR 0.80; 95% CI 0.72–0.89) for GLP‐1 RAs as a class, with the SELECT trial contributing 77% of the analysis weight [10]. The meta‐analysis also demonstrated reductions in stroke (RR 0.72), myocardial infarction (RR 0.84), and heart failure hospitalization (RR 0.82), with a mediation analysis suggesting that 35%–55% of cardiovascular benefit was independent of weight reduction.

Several factors may explain the apparent cardiovascular‐superiority of tirzepatide in T2D but of semaglutide in non‐T2D: (1) channeling bias, whereby patients perceived as higher cardiovascular risk may be preferentially prescribed semaglutide (which had earlier evidence of benefit in cardiovascular outcome trials) rather than tirzepatide; (2) differential follow‐up duration, with tirzepatide having shorter post‐marketing exposure; (3) diabetes status as a genuine effect modifier of the comparative cardiovascular efficacy of these agents; and (4) residual confounding inherent to all observational comparisons. The magnitude of some real‐world cardiovascular effect sizes (e.g., HR 0.54 for MACE) substantially exceeds RCT estimates (RR 0.80), suggesting that residual confounding or healthy‐user bias may inflate real‐world estimates.

### 3.5 Other Outcomes

Beyond metabolic and cardiovascular end‐points, real‐world studies have identified potentially important effects across multiple organ systems, though these findings are largely exploratory.

Sarcopenia: We have commented previously on the potential that incretin receptor activators may be associated with clinically important skeletal muscle loss in a subset of treated persons, an issue which may particularly be encountered in older persons receiving these agents, who are likely to have greater degrees of frailty and more comorbidities than those of participants in clinical trials; assessment of walking speed and of grip strength and measurement of the ratio of serum creatinine to cystatin C may be useful approaches to assessment of sarcopenia [36].

Pulmonary outcomes: In a large propensity‐score matched study of 331 863 patients with T2D, Henney et al. found that GLP‐1 RA use (versus DPP‐4 inhibitor use) was associated with markedly lower risks of pneumonia (HR 0.60; 95% CI 0.58–0.62) and sepsis (HR 0.61; 95% CI 0.59–0.63) [37]. Whether this reflects anti‐inflammatory properties of GLP‐1 RAs, reduced aspiration risk through weight loss and delayed gastric emptying, or residual confounding requires further investigation.

Neurological outcomes: Schechter et al. (*n* = 214 442, T2D) reported a composite neurodegeneration hazard ratio of 0.81 (95% CI 0.77–0.86) with GLP‐1 RA use [38]. Multiple studies by Wang et al. found that GLP‐1 RAs were associated with substantially lower Alzheimer's disease risk in T2D (HR 0.33–0.59 versus insulin; HR 0.54–0.80 versus other glucose‐lowering agents) [39]. Although the evoke/evoke+ phase 3 trials failed to show an effect of oral semaglutide on functional and cognitive decline in early‐stage symptomatic Alzheimer's disease, nearly half of participants had BMI < 25 [40], more recent real‐world and target trial emulation evidence—including a propensity‐matched cohort study, analyses of TriNetX and national EHR databases, and a target trial emulation in T2D—supports GLP‐1 RA‐associated reductions in Alzheimer's disease risk [41, 42, 43].

Psychiatric outcomes: Contrary to early regulatory signals of concern, Wang et al. found that semaglutide was associated with markedly lower risk of incident suicidal ideation (HR 0.27; 95% CI 0.20–0.36) in a large cohort of 240 618 individuals with obesity, replicated in a T2D cohort of 1 589 855 [44]., and in our analysis of a population of ~8 million persons with T2D [45], semaglutide similarly reduced risk of suicidal ideation. Semaglutide was also associated with 50%–56% lower risk of alcohol use disorder in separate analyses [46].

Safety and persistence: Thomsen et al. summarized real‐world discontinuation rates at 20%–50% within 1 year, compared with approximately 10% in RCTs—a difference that substantially affects intention‐to‐treat effectiveness [8]. Gastrointestinal adverse events remain the primary driver of discontinuation. Reassuringly, real‐world data have not confirmed hypothesized risks of pancreatitis, thyroid cancer, or depression. The Vienghirun NMA reported higher nausea and vomiting with tirzepatide (RR 3.64; 95% CI 2.40–5.52 versus placebo), consistent with its more potent weight‐loss effect [11].

## 4 Discussion

**FIGURE 1:**
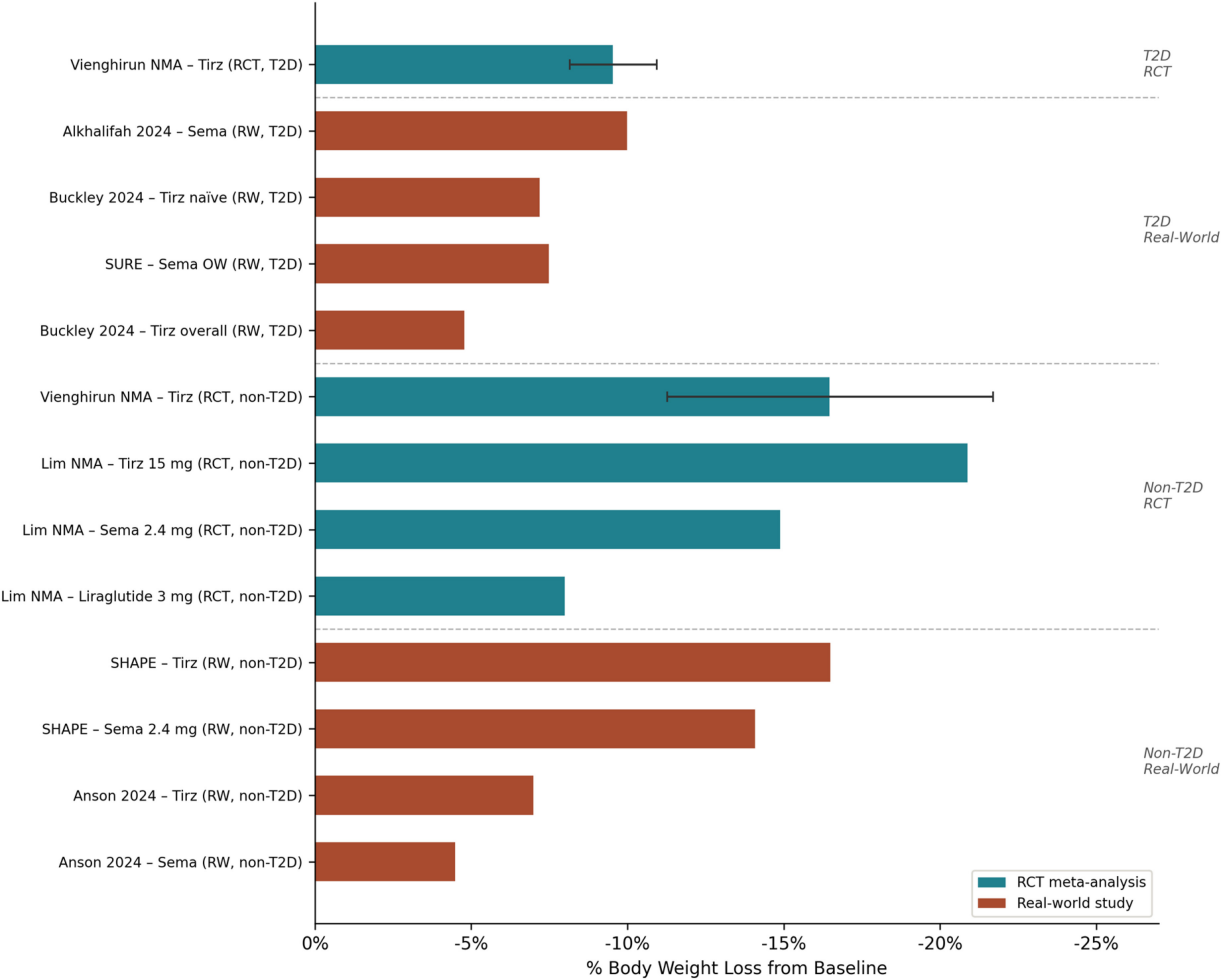
Percentage body weight loss with semaglutide 2.4 mg and tirzepatide in RCT meta‐analyses vs. real‐world studies, by diabetes status. Horizontal bars represent point estimates of percentage body weight reduction from baseline. Blue‐green bars indicate RCT meta‐analytic estimates (placebo‐adjusted where applicable); red bars indicate real‐world estimates. Error bars represent 95% confidence intervals where reported. Studies are grouped by population (type 2 diabetes [T2D] and non‐T2D) and study type (RCT meta‐analysis vs. real‐world). Real‐world persister analyses approximate per‐protocol RCT estimates, whereas intention‐to‐treat real‐world analyses show substantially lower weight loss. Sources: Lim et al. 2026; Vienghirun et al. 2026; Ng et al. 2025/SHAPE; Anson et al. 2024; Wolffenbuttel et al. 2023; Buckley et al. 2024; Alkhalifah et al. 2024.

This narrative review compares seven 2026 RCT meta‐analyses with 26 real‐world studies of incretin‐based therapies, finding RCTs to show comparable effects to those of real‐world studies in weight loss among people having and not having T2D, in HbA1c among people with T2D, and in MACE (Table 6). Real‐world effectiveness is broadly consistent with RCT efficacy, with both RCT and real‐world weight loss among people with diabetes being less than that among people not having diabetes (Figure 1). The findings have direct implications for clinical practice, health system planning, and future research priorities.

**TABLE 6:** Summary comparison: RCT vs. real‐world effectiveness.

Outcome	Drug	RCT estimate	RW estimate	Gap direction	RW comments
Wt loss %, T2D	Tirzepatide	−9.55% (NMA)	−4.8% to −7.2%	RW < RCT	Sub‐max dosing; prior GLP‐1RA
Wt loss %, T2D	Semaglutide	~5%–6% (NMA, SC)	−7.5% to −10%	RW ≈ or > RCT	Comparable; 12‐months studies capture durability
Wt loss %, non‐T2D	Tirzepatide	−16.5% to −20.9%	−16.5% (persisters); ~7% (ITT)	Persisters ≈ RCT; ITT << RCT	Only 25.9% reach 15 mg; discontinuation
Wt loss %, non‐T2D	Sema 2.4 mg	−14.9%	−14.1% (persisters); ~4.5% (ITT)	Persisters ≈ RCT; ITT << RCT	13% reach 2.4 mg; 20%–50% d/c rate
HbA1c, T2D	Semaglutide	−1.0% to −1.8%‐pts	−1.0% to −1.4%‐patients	RW ≈ RCT (lower end)	Glycemic control less dose‐dependent
HbA1c, T2D	Tirzepatide	−1.5% to −2.3%‐pts	−0.6% to −1.0%‐patients	RW < RCT	Prior GLP‐1RA exposure; sub‐max dose
MACE, T2D	Tirz vs. Sema	No head‐to‐head CVOT	HR 0.54–0.86	Tirz may be superior	Possible channeling bias; short follow‐up
MACE, non‐T2D	GLP‐1 RA class	RR 0.80 (0.72–0.89)	HR 0.71 (STEER, sema vs. tirz)	Broadly consistent	Different comparators (PBO vs. active)

*Note:* See Tables 2, 3, 4, 5 for other abbreviations.

Abbreviations: CVOT, cardiovascular outcome trial; d/c, discontinuation; ITT, intention‐to‐treat; RW, real‐world.

The most striking finding is the concordance between real‐world persister estimates and per‐protocol RCT estimates for weight loss in non‐diabetic populations. In the SHAPE study, persistent semaglutide users achieved 14.1% weight loss at 12 months—closely mirroring the 14.9% placebo‐adjusted estimate from the Lim NMA—while persistent tirzepatide users achieved 16.5%, consistent with the expected dose‐weighted average given that only 25.9% reached 15 mg (Lim NMA: 17.8% at 10 mg, 20.9% at 15 mg) [12, 24]. This concordance suggests that incretin‐based therapies are genuinely as effective in real‐world patients as in trial participants, provided that patients persist with treatment and achieve adequate doses. The clinical implication: “real‐world discount” is not a property of the drugs themselves but of adherence issues.

**FIGURE 2:**
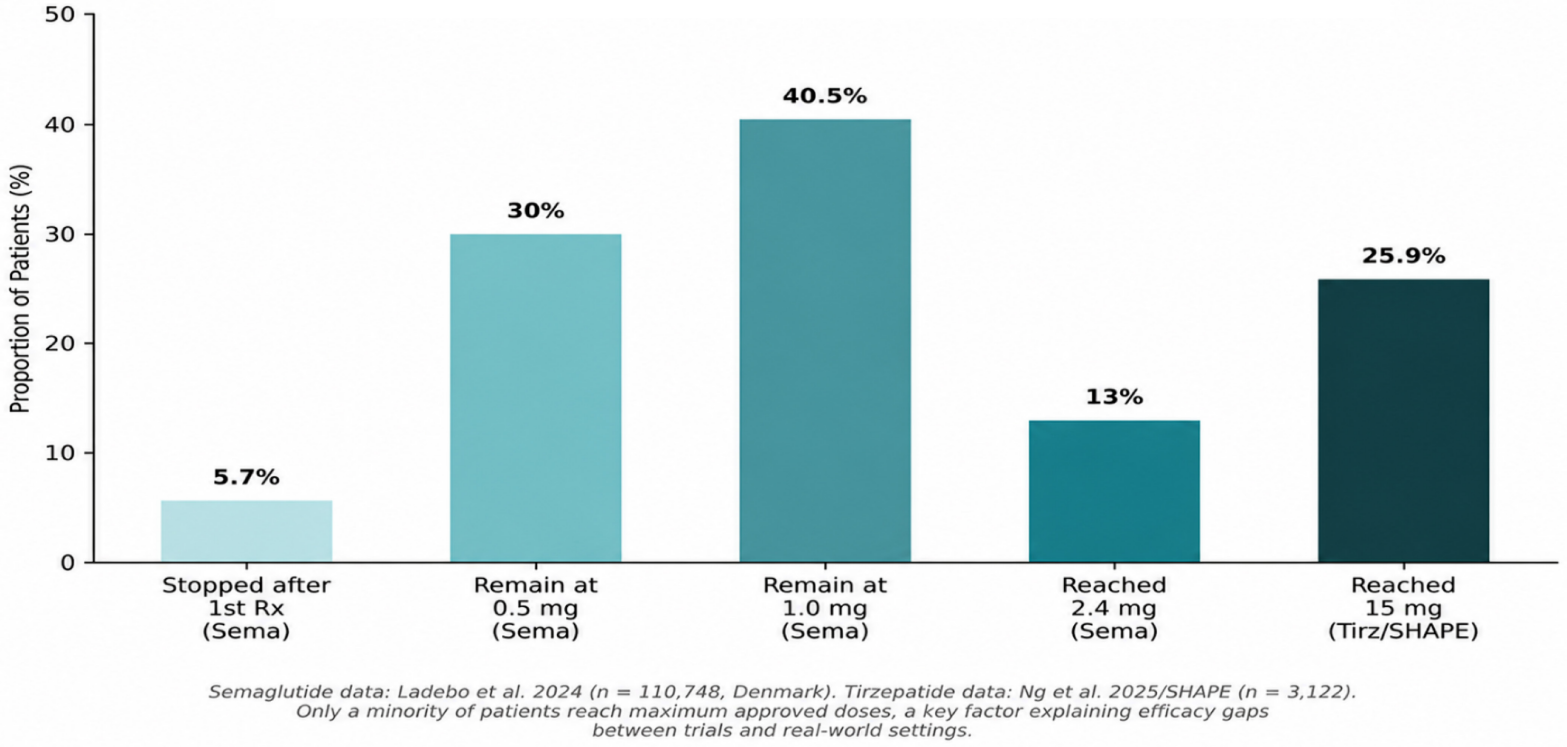
Proportion of patients reaching target doses in real‐world practice. Semaglutide data from Ladebo et al. 2024 (*n* = 110 748, Denmark); tirzepatide data from Ng et al. 2025/SHAPE (*n* = 3122). Among semaglutide users, 5.7% discontinued after the first prescription, approximately 30% remained at 0.5 mg, 33%–48% remained at 1.0 mg from the fourth prescription onward, and only 13% reached the maximum approved dose of 2.4 mg. Among tirzepatide users, 25.9% reached 15 mg. The low proportion of patients reaching maximum doses is a key factor explaining the efficacy gap between RCTs (where doses are systematically titrated to target) and real‐world settings.

Sub‐maximal dose titration emerges as the single most actionable explanation for the efficacy–effectiveness gap (Figure 2). In the Ladebo Danish registry, only 13% of semaglutide users reached 2.4 mg, with the majority stabilizing at 1.0 mg [7]. This is not merely a matter of tolerability: it reflects real‐world prescribing patterns, insurance coverage limitations, supply constraints, and variable clinical ambition for weight loss. The dose–response relationship for incretin‐based therapies is steep; the difference between semaglutide 1.0 mg and 2.4 mg, or tirzepatide 5 mg and 15 mg, represents a doubling or tripling of expected weight loss. Structured dose titration protocols with explicit targets, patient education about the dose–response relationship, and systematic barriers removal (including prior authorization reform) are likely the highest‐yield interventions to close the efficacy–effectiveness gap.

The cardiovascular data present a paradox that warrants careful interpretation. In T2D populations, two large real‐world studies found tirzepatide to be cardiovascular‐superior to semaglutide (Anson 2024: HR 0.54 for composite cardiovascular events; Henney 2025: HR 0.86 for MACE in T2D with obstructive sleep apnea) [25, 27]. In non‐T2D populations, however, the STEER study found semaglutide cardiovascular‐superior to tirzepatide (HR 0.71 for recurrent MACE‐3) [35]. While this divergence could reflect a genuine diabetes‐status‐dependent difference in comparative cardiovascular efficacy, channeling bias is a more parsimonious explanation: clinicians may preferentially prescribe semaglutide to higher‐risk non‐diabetic patients based on SELECT trial data, whereas tirzepatide may be channeled to higher‐risk T2D patients based on SURPASS glycemic superiority data. Moreover, some real‐world cardiovascular hazard ratios (HR 0.54) substantially exceed the 20% MACE reduction observed in the SELECT trial (RR 0.80) [6, 10], Importantly, SURPASS‐CVOT data confirm tirzepatide's cardiovascular benefit versus dulaglutide in T2D [28], and an imputed placebo analysis estimates tirzepatide's MACE reduction to be approximately 20% versus placebo, comparable to semaglutide's SELECT estimate [30]. Real‐world evidence from a large active‐comparator study (*n* = 113 209) found no significant MACE difference between semaglutide and tirzepatide in T2D (HR 0.97; 95% CI 0.88–1.07), consistent with class‐level rather than drug‐specific benefit [34] suggesting that residual confounding inflates real‐world estimates despite propensity‐score matching.

Industry sponsorship pervades the evidence base for incretin‐based therapies. Alexander et al. found that 96.2% of RCTs of GLP‐1 RAs for weight loss were industry‐funded [9]. The Ciudin Bayesian NMA, funded by Eli Lilly (tirzepatide manufacturer), excluded SURMOUNT‐5—the only head‐to‐head RCT of tirzepatide versus semaglutide at maximum tolerated doses, which favored tirzepatide by approximately 5.5 percentage points—on the grounds that it used maximum tolerated rather than fixed doses [13]. While this exclusion has methodological justification, it removes the most clinically relevant comparison. These observations do not invalidate the existing evidence but emphasize the need for independent replication, particularly for comparative effectiveness claims.

Several important limitations of this analysis must be acknowledged. First, most real‐world studies had follow‐up of 12 months or less, precluding assessment of long‐term durability, safety signals, or cancer risk. Second, no randomized evidence exists for cognitive, neurological, or psychiatric end‐points; the reported hazard ratios for neurodegeneration and suicidal ideation, however provocative, arise from observational designs susceptible to healthy‐user bias and unmeasured confounding. Third, the observational cardiovascular data compare different drug pairings across different populations with different comparators, making cross‐study inference hazardous. Fourth, in lieu of a formal quality assessment using validated tools (e.g., Newcastle‐Ottawa Scale), pragmatic evidence quality categorization was utilized.

The type 1 diabetes data merit specific comment. The Mirghani meta‐analysis showed that GLP‐1 RAs produce modest HbA1c reduction (−0.4 percentage points) and more substantial weight loss (−4.28 kg) in T1D without increasing hypoglycemia, but with more adverse events [15]. Furthermore, real‐world T1D data from Anson et al. suggest that SGLT2 inhibitors may be preferable for cardiorenal protection, given the lower heart failure and chronic kidney disease risk observed with these agents in comparison with GLP‐1 RAs [47]. Off‐label GLP‐1 RA use in T1D should therefore be cautious and should not substitute for evidence‐based cardiorenal agents, while recognizing the potential issue of SGLT2 inhibitor‐induced ketoacidosis [48].

Several future research directions are indicated. First, longer real‐world follow‐up (3–5 years) is needed to assess weight loss durability, weight regain after discontinuation, and rare safety signals. Second, sex‐stratified real‐world analyses should be conducted to determine whether the 4.1 percentage‐point sex difference in weight loss identified by Alexander et al. persists outside of trials [9]. Third, implementation of structured dose titration protocols could directly address the most actionable driver of the efficacy–effectiveness gap. Fourth, further randomized trials of incretin‐based therapies with cognitive decline as a primary endpoint are warranted given the consistent observational signal, using populations with greater prevalence of overweight and obesity to optimize the potential that GLP‐1 RAs act particularly in this group.

In conclusion, the evidence synthesized in this review demonstrates that incretin‐based therapies deliver real‐world effectiveness consistent with RCT efficacy in patients who achieve and maintain target doses. The primary drivers of the efficacy–effectiveness gap—sub‐maximal dosing, early discontinuation, and prior GLP‐1 RA exposure—are modifiable through clinical and health system interventions. Clinicians counseling patients can communicate that RCT‐level outcomes are achievable with adequate dose titration and persistence, while acknowledging that population‐level outcomes will be lower due to the realities of routine clinical practice.

## Funding

The author has nothing to report.

## Ethics Statement

This manuscript is a narrative review based on published literature. No human participants, animals, or identifiable patient data were involved; therefore, ethics committee approval and informed consent were not required.

## Conflicts of Interest

The author declares no conflicts of interest.

## Data Availability

The data used to support this study is all derived from the cited published literature.
